# A case study of an individual participant data meta-analysis of diagnostic accuracy showed that prediction regions represented heterogeneity well

**DOI:** 10.1038/s41598-023-36129-w

**Published:** 2023-06-07

**Authors:** Aurelio López Malo Vázquez de Lara, Parash Mani Bhandari, Yin Wu, Brooke Levis, Brett Thombs, Andrea Benedetti, DEPRESsion Screening Data (DEPRESSD) PHQ-9 Collaboration, Ying Sun, Chen He, Ankur Krishnan, Dipika Neupane, Zelalem Negeri, Mahrukh Imran, Danielle B. Rice, Kira E. Riehm, Nazanin Saadat, Marleine Azar, Jill Boruff, Pim Cuijpers, Simon Gilbody, John P. A. Ioannidis, Lorie A. Kloda, Dean McMillan, Scott B. Patten, Ian Shrier, Roy C. Ziegelstein, Dickens H. Akena, Bruce Arroll, Liat Ayalon, Hamid R. Baradaran, Anna Beraldi, Charles H. Bombardier, Peter Butterworth, Gregory Carter, Marcos H. Chagas, Juliana C. N. Chan, Rushina Cholera, Neerja Chowdhary, Kerrie Clover, Yeates Conwell, Janneke M. de Man-van Ginkel, Jaime Delgadillo, Jesse R. Fann, Felix H. Fischer, Daniel Fung, Bizu Gelaye, Felicity Goodyear-Smith, Catherine G. Greeno, Brian J. Hall, Martin Härter, Ulrich Hegerl, Leanne Hides, Stevan E. Hobfoll, Marie Hudson, Thomas Hyphantis, Masatoshi Inagaki, Khalida Ismail, Nathalie Jetté, Mohammad E. Khamseh, Kim M. Kiely, Yunxin Kwan, Femke Lamers, Shen-Ing Liu, Manote Lotrakul, Sonia R. Loureiro, Bernd Löwe, Laura Marsh, Anthony McGuire, Sherina Mohd Sidik, Tiago N. Munhoz, Kumiko Muramatsu, Flávia L. Osório, Vikram Patel, Brian W. Pence, Philippe Persoons, Angelo Picardi, Katrin Reuter, Alasdair G. Rooney, Iná S. Santos, Juwita Shaaban, Abbey Sidebottom, Adam Simning, Lesley Stafford, Sharon C. Sung, Pei Lin Lynnette Tan, Alyna Turner, Christina M. van der Feltz-Cornelis, Henk C. van Weert, Paul A. Vöhringer, Jennifer White, Mary A. Whooley, Kirsty Winkley, Mitsuhiko Yamada, Yuying Zhang

**Affiliations:** 1 Lady Davis Institute for Medical Research, Jewish General Hospital, Montreal, QC Canada; 2 Department of Epidemiology, Biostatistics and Occupational Health, McGill University, Montreal, QC Canada; 3 Department of Psychiatry, McGill University, Montreal, QC Canada; 4 Centre for Prognosis Research, School of Primary, Community and Social Care, Keele University, Staffordshire, UK; 5 Department of Medicine, McGill University, Montreal, QC Canada; 6 Department of Psychology, McGill University, Montreal, QC Canada; 7 Department of Educational and Counselling Psychology, McGill University, Montreal, QC Canada; 8 Biomedical Ethics Unit, McGill University, Montreal, QC Canada; 9 Respiratory Epidemiology and Clinical Research Unit, McGill University Health Centre, Montreal, QC Canada; 10 Centre for Outcomes Research & Evaluation, Research Institute of the McGill University Health Centre, 5252 Boulevard de Maisonneuve, Montreal, QC H4A 3S5 Canada; 11 Schulich Library of Physical Sciences, Life Sciences, and Engineering, McGill University, Montreal, QC Canada; 12 Department of Clinical, Neuro and Developmental Psychology, Amsterdam Public Health Research Institute, Vrije Universiteit Amsterdam, Amsterdam, The Netherlands; 13 Hull York Medical School and the Department of Health Sciences, University of York, Heslington, York UK; 14 Department of Medicine, Department of Epidemiology and Population Health, Department of Biomedical Data Science, Department of Statistics, Stanford University, Stanford, CA USA; 15 Library, Concordia University, Montreal, QC Canada; 16 Department of Community Health Sciences, University of Calgary, Calgary, AB Canada; 17 Department of Medicine, Johns Hopkins University School of Medicine, Baltimore, MD USA; 18 Department of Psychiatry, Makerere University College of Health Sciences, Kampala, Uganda; 19 Department of General Practice and Primary Health Care, University of Auckland, Auckland, New Zealand; 20 Louis and Gabi Weisfeld School of Social Work, Bar Ilan University, Ramat Gan, Israel; 21 Endocrine Research Center, Institute of Endocrinology and Metabolism, Iran University of Medical Sciences, Tehran, Iran; 22 Kbo-Lech-Mangfall-Klinik Garmisch-Partenkirchen, Klinik Für PsychiatriePsychotherapie & Psychosomatik, Lehrkrankenhaus der Technischen Universität München, Munich, Germany; 23 Department of Rehabilitation Medicine, University of Washington, Seattle, WA USA; 24 Centre for Research on Ageing, Health and Wellbeing, Research School of Population Health, The Australian National University, Canberra, Australia; 25 Centre for Brain and Mental Health Research, University of Newcastle, Newcastle, NSW Australia; 26 Department of Neurosciences and Behavior, Ribeirão Preto Medical School, University of São Paulo, Ribeirão Preto, Brazil; 27 Department of Medicine and Therapeutics, Prince of Wales Hospital, The Chinese University of Hong Kong, Ma Liu Shui, Hong Kong Special Administrative Region China; 28 Department of Pediatrics, Duke University, Durham, NC USA; 29 Clinical Psychiatrist, Mumbai, India; 30 Department of Psychiatry, University of Rochester Medical Center, Rochester, NY USA; 31 Leids Univesity Medical Center, Leiden, The Netherlands; 32 Clinical Psychology Unit, Department of Psychology, University of Sheffield, Sheffield, UK; 33 Department of Psychiatry and Behavioral Sciences, University of Washington, Seattle, WA USA; 34 Department of Psycosomatic Medicine, Center for Internal Medicine and Dermatology, Charité - Universitätsmedizin Berlin, Berlin, Germany; 35 Department of Developmental Psychiatry, Institute of Mental Health, Singapore, Singapore; 36 Department of Epidemiology, Harvard T. H. Chan School of Public Health, Boston, MA USA; 37 School of Social Work, University of Pittsburgh, Pittsburgh, PA USA; 38 Center for Global Health Equity, New York University Shanghai, Shanghai, People’s Republic of China; 39 Department of Medical Psychology, University Medical Center Hamburg-Eppendorf, Hamburg, Germany; 40 Department of Psychiatry, Psychosomatics and Psychotherapy, Goethe-Universität, Frankfurt, Germany; 41 School of Psychology, University of Queensland, Brisbane, QLD Australia; 42 STAR-Stress, Anxiety and Resilience Consultants, Chicago, IL USA; 43 Department of Psychiatry, Faculty of Medicine, School of Health Sciences, University of Ioannina, Ioannina, Greece; 44 Department of Psychiatry, Faculty of Medicine, Shimane University, Shimane, Japan; 45 Department of Psychological Medicine, Institute of Psychiatry, Psychology and Neurosciences, King’s College London Weston Education Centre, London, UK; 46 Department of Neurology, Icahn School of Medicine at Mount Sinai, New York, NY USA; 47 School of Psychology, The University of New South Wales, and Neuroscience Research Australia (NeuRA), Sydney, Australia; 48 Department of Psychological Medicine, Tan Tock Seng Hospital, Singapore, Singapore; 49 Department of Psychiatry, Amsterdam Public Health Research Institute, Amsterdam UMC, Vrije Universiteit, Amsterdam, The Netherlands; 50 Programme in Health Services & Systems Research, Duke-NUS Medical School, Singapore, Singapore; 51 Department of Psychiatry, Faculty of Medicine, Ramathibodi Hospital, Mahidol University, Bangkok, Thailand; 52 Department of Psychosomatic Medicine and Psychotherapy, University Medical Center Hamburg-Eppendorf, Hamburg, Germany; 53 Baylor College of Medicine, Houston and Michael E. DeBakey Veterans Affairs Medical Center, Houston, TX USA; 54 College of Nursing, University of South Florida, Tampa, FL USA; 55 Department of Psychiatry, Faculty of Medicine and Health Sciences, Universiti Putra Malaysia, Serdang, Selangor Malaysia; 56 Post-Graduate Program in Epidemiology, Federal University of Pelotas, Pelotas, RS Brazil; 57 Niigata Seiryo University Health Service Center,, Niigata, Japan; 58 Department of Global Health and Social Medicine, Harvard Medical School, Boston, MA USA; 59 Department of Epidemiology, Gillings School of Global Public Health, The University of North Carolina at Chapel Hill, Chapel Hill, NC USA; 60 Department of Adult Psychiatry, University Hospitals Leuven, Leuven, Belgium; 61 Centre for Behavioural Sciences and Mental Health, Italian National Institute of Health, Rome, Italy; 62 Group Practice for Psychotherapy and Psycho-Oncology, Freiburg, Germany; 63 Division of Psychiatry, Royal Edinburgh Hospital, University of Edinburgh, Edinburgh, Scotland, UK; 64 Department of Family Medicine, School of Medical Sciences, Universiti Sains Malaysia, Kelantan, Malaysia; 65 Allina Health, Minneapolis, MN USA; 66 Centre for Women’s Mental Health, Royal Women’s Hospital, Parkville, Australia; 67 School of Medicine and Public Health, University of Newcastle, Newcastle, NSW Australia; 68 Department of Health Sciences, HYMS, University of York, York, UK; 69 Department General Practice, Institute Public Health, Amsterdam Universities Medical Centers, Amsterdam, The Netherlands; 70 Department of Psychiatry and Mental Health, Clinical Hospital, Universidad de Chile, Santiago, Chile; 71 Department of Physiotherapy, School of Primary and Allied Health Care, Monash University, Melbourne, Australia; 72 Department of Epidemiology and Biostatistics, University of California San Francisco, San Francisco, CA USA; 73 Florence Nightingale Faculty of Nursing, Midwifery & Palliative Care, King’s College London, London, UK; 74 Department of Neuropsychopharmacology, National Institute of Mental Health, National Center of Neurology and Psychiatry, Ogawa-Higashi, Kodaira, Tokyo Japan

**Keywords:** Psychology, Depression, Statistics

## Abstract

The diagnostic accuracy of a screening tool is often characterized by its sensitivity and specificity. An analysis of these measures must consider their intrinsic correlation. In the context of an individual participant data meta-analysis, heterogeneity is one of the main components of the analysis. When using a random-effects meta-analytic model, prediction regions provide deeper insight into the effect of heterogeneity on the variability of estimated accuracy measures across the entire studied population, not just the average. This study aimed to investigate heterogeneity via prediction regions in an individual participant data meta-analysis of the sensitivity and specificity of the Patient Health Questionnaire-9 for screening to detect major depression. From the total number of studies in the pool, four dates were selected containing roughly 25%, 50%, 75% and 100% of the total number of participants. A bivariate random-effects model was fitted to studies up to and including each of these dates to jointly estimate sensitivity and specificity. Two-dimensional prediction regions were plotted in ROC-space. Subgroup analyses were carried out on sex and age, regardless of the date of the study. The dataset comprised 17,436 participants from 58 primary studies of which 2322 (13.3%) presented cases of major depression. Point estimates of sensitivity and specificity did not differ importantly as more studies were added to the model. However, correlation of the measures increased. As expected, standard errors of the logit pooled TPR and FPR consistently decreased as more studies were used, while standard deviations of the random-effects did not decrease monotonically. Subgroup analysis by sex did not reveal important contributions for observed heterogeneity; however, the shape of the prediction regions differed. Subgroup analysis by age did not reveal meaningful contributions to the heterogeneity and the prediction regions were similar in shape. Prediction intervals and regions reveal previously unseen trends in a dataset. In the context of a meta-analysis of diagnostic test accuracy, prediction regions can display the range of accuracy measures in different populations and settings.

## Introduction

Medical screening tests are used to identify possible disease before signs or symptoms present, such as HIV antibody testing, or to identify the presence of a condition that has not otherwise been identified, such as in depression screening. The accuracy of a screening test is evaluated by comparing against a reference standard that is thought to represent the true status of the target condition. Accuracy is typically characterized by sensitivity or true positive rate (TPR), which is the probability of a positive screen given that the patient has the condition, and 1-specificity or false positive rate (FPR), which is the probability of a positive screen given that the patient does not have the condition. When screening test results are ordinal or continuous, a threshold is set to classify test results as positive or negative.

Meta-analyses of test accuracy pool results from primary studies to attempt to overcome imprecision due to small samples, conduct subgroup analyses that were not feasible in the primary studies, and estimate variance within and between studies^1^. Such meta-analyses must consider the intrinsic correlation between TPR and FPR across studies. This is because selecting a lower threshold for classifying positive screening results would simultaneously increase the TPR of the test but also its FPR while a higher threshold would have the opposite effect. The bivariate random effects model (BREM) is commonly used because it allows for simultaneous estimation of TPR and FPR with the random effects assumed to have a joint normal distribution^2^.

The inter-study heterogeneity or variability of TPR and FPR is an important output from a meta-analysis and may be characterized by several different metrics. The most direct is the between-study variance, often denoted as ${\tau }^{2}$. However, given that this parameter ranges from zero to infinity, interpreting its value as “small” or “large” is difficult. Other approaches to characterize heterogeneity, such as Cochran’s $Q$ or ${I}^{2}$ have been shown to have important limitations as well. Cochran’s $Q$ has limited power with small numbers of studies and is overly sensitive with large numbers of studies^3^, whereas ${I}^{2}$ ranges from 0 to 1 and represents the proportion of observed variability attributable to heterogeneity but does not provide information about variation in sensitivity or specificity^4^.

Another way to describe heterogeneity is the prediction interval. This represents an estimated range of values that has a predetermined probability of containing the estimate of interest from a new study sampled from the same population as used to fit the model. The use of prediction intervals has important advantages in that it summarizes point estimates and variance components from the BREM as the interval considers overall mean estimates of TPR and FPR, their standard errors and between-study variance^5^. The *Cochrane Handbook for Systematic Reviews of Diagnostic Test Accuracy* regards the prediction interval as the best graphical depiction of the magnitude of heterogeneity^6^. In the bivariate case, prediction intervals for TPR and FPR are represented as regions in two-dimensional space where this set of values has a predetermined probability of containing a new two-dimensional vector of estimates from a new study comparable to those in the pool^7^.

The Patient Health Questionnaire-9 (PHQ-9) is a self-report depression symptom questionnaire that consists of nine items, each scored 0 to 3 (total possible score 0 to 27), that can be used for depression screening. A standard threshold score of 10 or greater has been shown to maximize the sum of sensitivity and specificity (TPR and 1-FPR)^8^. Meta-analyses assessing the diagnostic accuracy of the PHQ-9 and other similar screening questionnaires often report a confidence interval around pooled estimates of TPR and FPR^9,10^. The confidence interval contains the true value of the mean measure, here the pooled estimate of TPR or FPR with probability 0.95. Consequently, other authors have argued that prediction intervals are more useful since they provide information on the range of possible accuracy values that may be encountered in a future study^11,12^. Thus, a prediction interval and a confidence interval are not the same thing and serve different purposes. The confidence interval is a measure of precision that indicates how accurately we have estimated the pooled sensitivity or specificity based on the standard error and depends on the number of studies in the meta-analysis^13^. On the other hand, the prediction interval measures dispersion, is based on the standard deviation that shows how much the measures vary across different populations, and is not related to the number of studies in the analysis^13^. The prediction interval is more informative when it comes to heterogeneity, describing the extent of dispersion in the context of sensitivity or specificity^13^. A clinician using the results from a meta-analysis on the diagnostic accuracy of the PHQ-9 would have a better idea of how the diagnostic accuracy varied across studies, and indeed, how the PHQ-9 might be expected to perform in a new study, or setting such as the physician’s practice.

Because diagnostic accuracy is represented by the true positive rate (TPR) and the false positive rate (FPR), a prediction region is used rather than two prediction intervals. The region, which is elliptical in the logit space, takes into account the correlation between logit(TPR) and logit(FPR) which is reflected in the orientation and size of the minor axes of the prediction ellipse. The orientation of the ellipse relates to the “slope” linking logit(TPR) and logit(FPR) observations. The strength of the correlation is depicted by the width of the ellipses about their minor axis. Moreover, the prediction region makes explicit that some combinations of TPR and FPR are unlikely, whereas the two intervals do not.

Despite several sources suggesting that prediction intervals be used to quantify and describe heterogeneity and the range of accuracy values, they are still underused^11,12^. The objective of the present study was to illustrate the use of prediction regions for TPR and FPR of the PHQ-9 as a numerical and graphical depiction of the heterogeneity in an individual participant meta-analysis (IPDMA) and investigate how these regions (1) change as more studies are included in the BREM, and (2) vary across subgroups.

## Methods

This study is a secondary analysis of an IPDMA. For the main IPDMA registered in PROSPERO (CRD42014010673), a protocol was published^14^ and results have been reported^8^. The present analysis extends the work specified in the protocol by characterizing heterogeneity in the study pool via prediction regions constructed from the BREM, and using the region to describe the range of likely mean measures of TPR and FPR from an unseen study similar to those in the pool (with probability 0.95).

### Description of dataset

For the original IPDMA, studies were eligible for inclusion if: (1) they included PHQ-9 scores, (2) they included major depression classification based on a validated diagnostic interview, (3) the time interval between administration of the PHQ-9 and the diagnostic interview was no more than 2 weeks, and (4) participants were at least 18-years old and recruited outside of psychiatric settings. The studies and data included in the dataset were selected from the results of an online search strategy from 2000 to 2016. Eligible studies were assessed independently by two investigators. For further details on the search and selection processes, refer to the published protocol^14^.

### Data analysis for the present study

For each study in the PHQ-9 IPDMA dataset, generalized linear models were fitted to estimate TPR and FPR and their respective 95% CIs. From these, forest plots were produced for a qualitative assessment of heterogeneity. All analyses were completed in R^15^.

From the dataset, three dates were selected as “cutoff dates” (as reported in the “Date” column in Additional File 1). This approach was chosen to simulate how in reality more information becomes available on the topic over time and investigate the effects this accrual has on the heterogeneity of the study pool. The cutoff dates were selected so that participants in studies conducted up to and including each cutoff date comprised roughly 25%, 50% and 75% of the total number of participants. A BREM, as described in Additional File 2, was fitted for studies conducted up to and including each of the cutoff dates to jointly estimate TPR and FPR using the function “glmer” from the package “lme4”^15,16^. As these measures are described in two-dimensional ROC-space, confidence and prediction regions are analyzed instead of their one-dimensional analogues: confidence, and prediction intervals. For each model, 95% confidence and prediction regions were constructed following the method described by Chew^7^ (for more details, refer to Additional File 2). At each cutoff date, the confidence region associated with the model was plotted as well as the individual measure estimates from the studies used for the fit. Similarly, prediction regions were plotted with the individual measure estimates of studies after the corresponding cutoff date to assess coverage. Finally, a BREM was fitted using data from all studies and 95% confidence and prediction regions were constructed in the same manner as above. To quantify the size of all prediction regions, we also estimated the area of the interval in logit space.

Prediction intervals for participant subgroups were also constructed to assess whether heterogeneity could be due to age or sex or participants. Subgroups were defined using binary sex categories and, separately, age quartiles.

### Ethics approval and consent to participate

As this study involved only analysis of previously collected de-identified data and because all included studies were required to have obtained ethics approval and informed consent, the Research Ethics Committee of the Jewish General Hospital determined that ethics approval was not required.

## Results

The final IPDMA dataset consisted of 58 primary studies, totaling 17,436 participants of which 2322 (13.3%) presented cases of major depression and 1794 (10.3% of total, 77.3% of cases) were correctly identified as cases using the standard PHQ-9 cutoff score of ≥ 10.

### Main analysis

**Figure 1:**
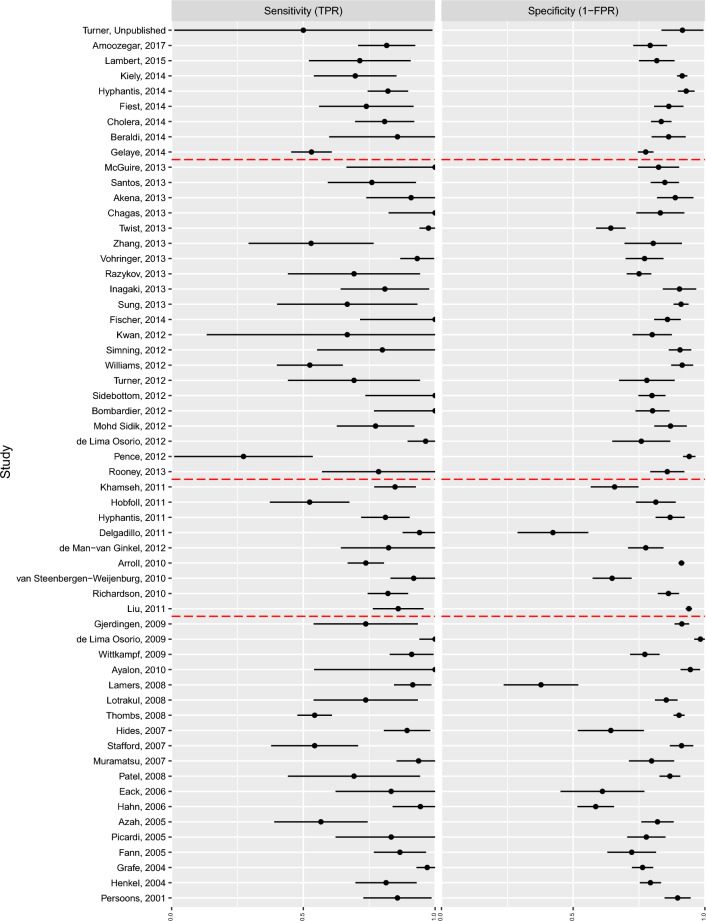
Forest plots of sensitivity (TPR) and specificity (1-FPR). (1) The dotted lines in the sensitivity forest plots indicate that the data from the study indicated a 100% true positive rate and a 0% false positive rate. This caused the sensitivity estimate to be 1 but the standard error was large enough to cover the whole interval $(0, 1)$. (2) Red dot-dashed lines indicate the selected cutoff dates for the BREM. (3) Studies are sorted by the year in which the study started, while the label indicates the year in which they were published.)

A summary of the individual participant data can be found in Additional File 1. The results of the generalized linear models per study for sensitivity and specificity (TPR and 1-FPR) can be seen in forest plots (Fig. 1). The presence of heterogeneity can be visually assessed in Fig. 1 Confidence intervals for specificity were much narrower than those for sensitivity.

**Figure 2:**
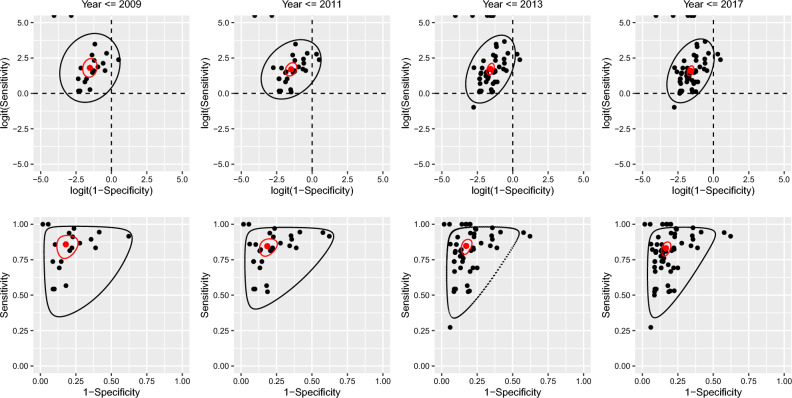
Prediction and confidence regions through time (FPR = 1 − specificity, TPR = sensitivity). Black dots are the study specific estimates. The red dot is the pooled estimate. The black line indicates the prediction region. The red line is the confidence region. The top panel show the estimates and regions in logit-space, while the bottom panel are in the probability-space. (1) The change in the estimated correlation between sensitivity and 1 − specificity is reflected in the orientation and size of the minor axes of the prediction ellipses in the top row of figure. The orientation of the ellipses relates to the “slope” linking TPR and FPR observations. The strength of the correlation is depicted by the width of the ellipses about their minor axis.)

Studies published up to and including the cutoff dates of 2009, 2011 and 2013 included 19, 28 and 49 of the 58 available studies and 28.2%, 59.4% and 81.8% of total participants respectively. At the 2009 cutoff, the TPR and FPR were 0.86 and 0.18 (see Table 1). Increasing the number of studies at each cutoff date did not result in important differences in the pooled TPR and FPR, and the 95% confidence intervals for their estimates overlapped. As the number of studies increased, the standard error of the estimated pooled TPR and FPR decreased (not shown), and correspondingly, the confidence intervals and regions shrank as expected (see Table 1 and Fig. 2).

**Table 1:** Summary of pooled FPR and TPR results from the BREM.

Time interval	Parameter	Estimate	95% CI
2001–2009	FPR (1 − specificity)	0.18	(0.12, 0.25)
	TPR (sensitivity)	0.86	(0.78, 0.92)
2001–2011	FPR (1 − specificity)	0.19	(0.14, 0.24)
	TPR (sensitivity)	0.85	(0.79, 0.89)
2001–2013	FPR (1 − specificity)	0.17	(0.14, 0.21)
	TPR (sensitivity)	0.85	(0.80, 0.89)
2001–2017	FPR (1 − specificity)	0.17	(0.14, 0.19)
	TPR (sensitivity)	0.83	(0.79, 0.87)

There was some evidence that between-study standard deviation estimates for FPR decreased from ${\widehat{\tau }}_{0}=0.87$ at the 2009 cutoff date, to ${\widehat{\tau }}_{0}=0.69$ when all studies were included (see Table 2), though confidence intervals largely overlapped. This decrease in the estimated variance component is reflected in the narrowing of the prediction region in ROC space along the FPR direction (see Fig. 2C*,D*). Correspondingly, the area of the prediction region decreased as more data was included (Table 2). The correlation estimates for the random effects increased (from 0.16 to 0.43) as more studies were included.

**Table 2:** Summary of between study variances from the BREM*

Cutoff date	Parameter	Estimate	Correlation (${\widehat{\rho }}_{\tau }$)	Area of prediction region
2009	${\tau }_{0}$	0.87 (0.53, 1.18)	0.16 (− 0.46, 0.71)	22.83
	${\tau }_{1}$	0.99 (0.48, 1.42)		
2011	${\tau }_{0}$	0.87 (0.59, 1.08)	0.32 (− 0.16, 0.71)	16.65
	${\tau }_{1}$	0.85 (0.54, 1.17)		
2013	${\tau }_{0}$	0.71 (0.54, 0.88)	0.44 (0.13, 0.73)	13.51
	${\tau }_{1}$	0.98 (0.69, 1.26)		
Full	${\tau }_{0}$	0.69 (0.54, 0.82)	0.43 (0.12, 0.71)	11.94
	${\tau }_{1}$	0.92 (0.67, 1.16)		

*All estimates are on the logit scales. Confidence intervals for ${\tau }_{0}$, ${\tau }_{1}, and {\widehat{\rho }}_{\tau }$ were estimated using parametric bootstrap with 1000 replicates.

The change in the estimated correlation between logit(TPR) and logit(FPR) is reflected in the orientation and size of the minor axes of the prediction ellipses in the top row of Fig. 2. The orientation of the ellipses relates to the “slope” linking logit(TPR) and logit(FPR) observations. The strength of the correlation is depicted by the width of the ellipses about their minor axis.

### Subgroup analyses

**Figure 3:**
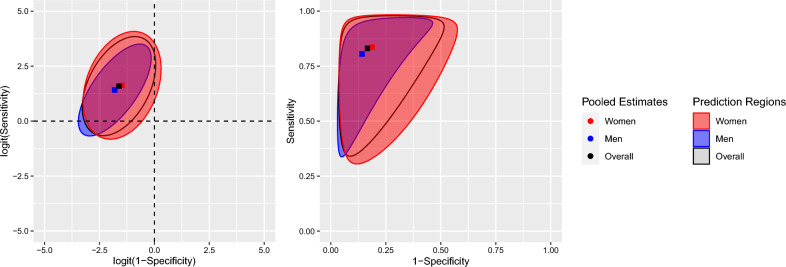
Prediction regions by sex subgroup (FPR = 1 − specificity, TPR = sensitivity).

Female participants represented 57% of participants. For this subgroup, BREM estimates for TPR and FPR were 0.84 and 0.19. For the male subjects, BREM estimates were 0.80 and 0.14 for TPR and FPR respectively (see Table 3). Between-study standard deviation estimates were both higher in the female group than in the male. Estimated correlation of the random effects was greater in the male group (see Table 3 and Fig. 3). The area of the region (in logit space) for the female subgroup was larger than that in the males (see Table 3 and Fig. 3). Overall, there was no clear indication that sex meaningfully contributes to heterogeneity in the whole sample.

**Table 3:** Summary of BREM by sex.

Subgroup	Parameter	Pooled estimate (95% CI)	Between study standard deviations* (95% CI)	${\widehat{\rho }}_{\tau }$ (95% CI)	Area of prediction region
Female	FPR	0.19 (0.16, 0.22)	0.73 (0.53, 0.88)	0.30 (− 0.08, 0.67)	14.58
	TPR	0.84 (0.79, 0.88)	1.00 (0.67, 1.29)		
Male	FPR	0.14 (0.12, 0.17)	0.68 (0.49, 0.83)	0.69 (0.35, 100)	9.03
	TPR	0.80 (0.75, 0.85)	0.85 (0.49, 1.15)		

*Standard deviations relate to the logit estimates. These are ${\tau }_{0}, {\tau }_{1.}$

**Figure 4:**
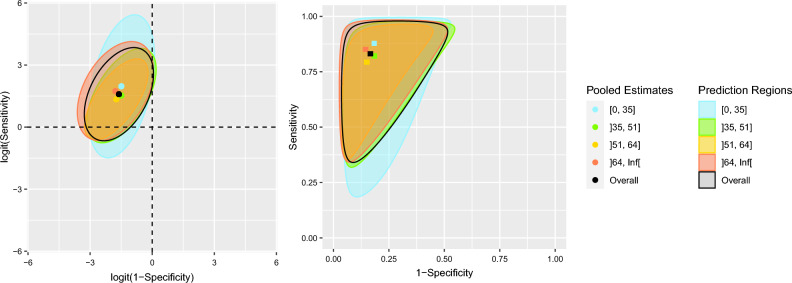
Prediction regions by age subgroup (FPR = 1 − specificity, TPR = sensitivity).

The quartiles for the age of participants were ${Q}_{1}=35$, ${Q}_{2}=51$, ${Q}_{3}=64$. Quantitative results of the BREM by age subgroup are presented in Table 4. Assessment of heterogeneity in these subgroups is summarized by the prediction regions in Fig. 4. In ROC space it can be observed that between-study standard deviation of FPR is not considerably different in any age subgroup ranging from 0.63 to 0.75. Between-study standard deviation of TPR is greatest among the group younger than 35 years (${\widehat{\tau }}_{1}=1.41$) and lowest for the group between 51 and 64 years old (${\widehat{\tau }}_{1}=0.80$). This comparison may also be observed by comparing the areas of their corresponding prediction regions. In logit-ROC space (Fig. 4A) it can be observed that the direction of correlation is similar across subgroups. The strength of the correlation, however, reaches a maximum in the age group between 51 and 64 years old (${\widehat{\rho }}_{\tau }=0.66$). No important contribution to the overall heterogeneity of the sample could be clearly identified from observing the prediction regions between the age subgroups.

**Table 4:** Summary of BREM by age subgroup.

Age range*	Parameter	Pooled estimate (95% CI)	Between study standard deviations* (95% CI)	${\widehat{\rho }}_{\tau }$ (95% CI)	Area of prediction region
$\left[0, 35\right]$	FPR	0.18 (0.15, 0.22)	0.66 (0.42, 0.86)	0.40 (− 0.08, 0.84)	18.31
	TPR	0.88 (0.80, 0.94)	1.41 (0.82, 2.02)		
$\left(35, 51\right]$	FPR	0.18 (0.15, 0.22)	0.68 (0.47, 0.86)	0.56 (0.15, 1.00)	11.43
	TPR	0.82 (0.76, 0.87)	0.93 (0.51, 1.30)		
$\left(51, 64\right]$	FPR	0.15 (0.12, 0.18)	0.63 (0.41, 0.80)	0.66 (0.20, 1.00)	8.24
	TPR	0.79 (0.73, 0.85)	0.80 (0.41, 1.14)		
$(64,\infty )$	FPR	0.14 (0.11, 0.17)	0.75 (0.49, 0.95)	0.42 (− 0.13, 1.00)	15.18
	TPR	0.85 (0.78, 0.92)	0.98 (0.42, 1.49)		

*The 25th, 50th and 75th percentiles were used to define the age ranges.

## Discussion

The present study aimed to characterize heterogeneity in an IPDMA of diagnostic test accuracy measures for the PHQ-9. The location of the overall estimates for TPR and FPR did not vary considerably as more studies were included. The size of the confidence region around the estimates shrank as more data were used in the model. The confidence region not only decreased in size but also changed shape as the correlation of the measures increased.

Prediction regions are one way to depict heterogeneity. Along the TPR axis, the prediction region changed erratically as more studies with differing estimates were included. Regarding FPR, the region consistently narrowed. This supports the initial inspection of the forest plots where sensitivity estimates (and therefore FPR), while scattered, showed less variability than the TPR estimates. The shape of the prediction region reflects the underlying positive correlation of these measures. Looking at Fig. 2D*, it can be observed that while a new study may estimate a high FPR (top-right corner of the prediction region), it is unlikely that the same study will simultaneously estimate a low TPR, say, below 0.5, as the coordinate in ROC-space would fall outside the 95% prediction region; this becomes more and more improbable as FPR increases. In the same way, a new study is unlikely to estimate low TPR and high FPR. The size of the prediction region is not guaranteed to decrease as more estimates are included, as seen in the 2013 cutoff (Fig. 2C*). The region updates, as more information becomes available on the location of individual estimates, to accurately represent the overall trends in the data.

Both confidence and prediction regions considering only the one-dimensional confidence/prediction intervals could be misleading, if interpreted naively. As an example, in Fig. 2D* the one-dimensional prediction intervals range from near 0 to almost 0.6 for FPR and from about 0.3 to 1 for TPR. However, if a clinician who administered the PHQ-9 wishes to consider the worst estimates for both accuracy measures i.e., 0.3 for TPR and 0.6 for FPR, this estimate is outside of the prediction region and the clinician could draw false conclusions from their assumptions.

The subgroup analyses aided in investigating possible sources of heterogeneity among the study pool. Prediction regions by subgroups can reveal some differences that might be hard to appreciate when only one-dimensional prediction intervals are used. Subgroup analysis by sex revealed no statistically significant differences between the point estimates of mean TPR and FPR between the female and male groups or when compared to overall population estimates; this coincides with the results of the main PHQ-9 IPDMA^8^. Both prediction regions for male and female groups span a comparable length parallel to either axis, although the shape of the ellipse in logit-ROC or the slanted border in ROC space differ between groups to some extent. This may be a depiction of the observed difference in the point estimates for correlation for both fixed and random effects between the female and male groups. The location of TPR and FPR estimates by age subgroup did not differ greatly from the overall population estimates. These regions were similar in size and shape: the largest region corresponding to the age group between 18 and 35 years old, being widest in the TPR direction, and the smallest corresponding to the 51 to 64 cohorts. While categorizing age has some downsides, it allowed us to present prediction regions by age category and improved interpretation of results.

The use of prediction intervals has been suggested in the literature as a complete summary of a random effects meta-analysis and a proper characterization of heterogeneity^17^. In meta-analyses that aim to estimate drug efficacy for a certain condition, prediction intervals provide information about its possible effects in a new, similar sample to the ones in the study pool. IntHout, Ioannidis, Rovers and Goeman report that prediction intervals which include the null value suggest that intervention effects could be null or even in the opposite direction of the intended effect^11^. In a meta-analysis, reporting only a confidence interval around an overall pooled estimate may mask the possibility that, in some setting, treatments are ineffective^12^. In the context of diagnostic accuracy, both TPR and FPR are sought to be different than 0.5. If either measure were to take on this value, the test would distinguish cases of major depression no better than a coin flip. Based on the prediction intervals in this study, it seems unlikely that in settings similar to the ones in the 58 studies available, both TPR and FPR are equal to 0.5. Prediction intervals have been reported to also aid in drawing conclusions from studies of varying size, instead of relying on the results of large studies^18^.

The present study had the advantage of access to a sizable data set of individual participant data collected from a large number of studies. The presence of heterogeneity was evident from preliminary analyses and later corroborated by graphical inspection of the prediction regions.

## Conclusions

The use of prediction regions allowed us to shed light regarding previously unseen trends in the data. In the present analysis, the varying correlations between TPR and FPR as more studies were added to the model and across subgroups were of special interest as they had noticeable effects on the shape of the prediction regions. The present analysis used prediction regions to investigate heterogeneity in the study pool and revealed greater heterogeneity regarding TPR estimates as compared to FPR estimates. Prediction regions display the full range of variability in the data, which is essential for making predictions, and uncovering trends which may have been otherwise unknown to the researcher; thus, supporting the recommendation by authors of using prediction regions as the most adequate summary of the results of a meta-analysis.

## Supplementary Information

Supplementary Tables.

Supplementary Information.

## Data Availability

Requests to access data should be made to the corresponding author at andrea.benedetti@mcgill.ca.

## Abbreviations

BREM: Bivariate random effects model
PHQ-9: Patient health questionnaire-9
IPDMA: Individual participant data meta-analysis
FPR: False positive rate (1 − specificity)
TPR: True positive rate (sensitivity)
