# Supplementary material for: A case study of an individual participant data meta-analysis of diagnostic accuracy showed that prediction regions represented heterogeneity well
Source: Sci Rep. 2023 Jun 7;13:9275. doi: 10.1038/s41598-023-36129-w (PMC10247712; doi:10.1038/s41598-023-36129-w)
Supplement: Supplementary file 1 — Supplementary Tables. [file 41598_2023_36129_MOESM1_ESM.docx]

**Supplementary Table 1: Summary of individual participant data.**

| **Author, year of publication** | **Date** | **Participants** | **True positives** | **True Negatives** | **Sensitivity** | **Specificity** |
| --- | --- | --- | --- | --- | --- | --- |
| Persoons, 2001 | 2001 | 173 | 24 | 130 | 0.86 | 0.90 |
| Henkel, 2004 | 2004 | 430 | 35 | 307 | 0.81 | 0.79 |
| Grafe, 2004 | 2004 | 494 | 65 | 326 | 0.97 | 0.76 |
| Fann, 2005 | 2005 | 135 | 39 | 65 | 0.87 | 0.72 |
| Picardi, 2005 | 2005 | 138 | 10 | 98 | 0.83 | 0.78 |
| Azah, 2005 | 2005 | 180 | 17 | 123 | 0.57 | 0.82 |
| Hahn, 2006 | 2006 | 211 | 17 | 113 | 0.94 | 0.59 |
| Eack, 2006 | 2006 | 48 | 10 | 22 | 0.83 | 0.61 |
| Patel, 2008 | 2007 | 299 | 9 | 248 | 0.69 | 0.87 |
| Muramatsu, 2007 | 2007 | 116 | 30 | 67 | 0.94 | 0.80 |
| Stafford, 2007 | 2007 | 193 | 19 | 144 | 0.54 | 0.91 |
| Hides, 2007 | 2007 | 103 | 42 | 36 | 0.89 | 0.64 |
| Thombs, 2008 | 2008 | 1006 | 120 | 708 | 0.54 | 0.90 |
| Lotrakul, 2008 | 2008 | 278 | 14 | 221 | 0.74 | 0.85 |
| Lamers, 2008 | 2008 | 104 | 54 | 17 | 0.92 | 0.38 |
| Ayalon, 2010 | 2009 | 151 | 6 | 137 | 1.00 | 0.94 |
| Wittkampf, 2009 | 2009 | 260 | 41 | 166 | 0.91 | 0.77 |
| Osorio, 2009 | 2009 | 177 | 60 | 115 | 1.00 | 0.98 |
| Gjerdingen, 2009 | 2009 | 419 | 14 | 365 | 0.74 | 0.91 |
| Liu, 2011 | 2010 | 1532 | 43 | 1392 | 0.86 | 0.94 |
| Richardson, 2010 | 2010 | 377 | 78 | 243 | 0.82 | 0.86 |
| van Steenbergen-Weijenburg, 2010 | 2010 | 196 | 34 | 103 | 0.92 | 0.65 |
| Arroll, 2010 | 2010 | 2528 | 115 | 2161 | 0.74 | 0.91 |
| de Man-van Ginkel, 2012 | 2011 | 164 | 14 | 114 | 0.82 | 0.78 |
| Delgadillo, 2011 | 2011 | 103 | 48 | 22 | 0.94 | 0.42 |
| Hyphantis, 2011 | 2011 | 213 | 56 | 125 | 0.81 | 0.87 |
| Hobfoll, 2011 | 2011 | 144 | 22 | 83 | 0.52 | 0.81 |
| Khamseh, 2011 | 2011 | 184 | 67 | 69 | 0.85 | 0.66 |
| Rooney, 2013 | 2012 | 126 | 11 | 96 | 0.79 | 0.86 |
| Pence, 2012 | 2012 | 398 | 3 | 364 | 0.27 | 0.94 |
| Osorio, 2012 | 2012 | 86 | 27 | 44 | 0.96 | 0.76 |
| Mohd Sidik, 2012 | 2012 | 146 | 24 | 100 | 0.77 | 0.87 |
| Bombardier, 2012 | 2012 | 160 | 14 | 117 | 1.00 | 0.80 |
| Sidebottom, 2012 | 2012 | 246 | 12 | 187 | 1.00 | 0.80 |
| Turner, 2012 | 2012 | 72 | 9 | 46 | 0.69 | 0.78 |
| Williams, 2012 | 2012 | 235 | 32 | 159 | 0.52 | 0.91 |
| Simning, 2012 | 2012 | 190 | 8 | 163 | 0.80 | 0.91 |
| Kwan, 2012 | 2012 | 113 | 2 | 88 | 0.67 | 0.80 |
| Fischer, 2014 | 2013 | 194 | 11 | 157 | 1.00 | 0.86 |
| Sung, 2013 | 2013 | 399 | 8 | 352 | 0.67 | 0.91 |
| Inagaki, 2013 | 2013 | 104 | 17 | 75 | 0.81 | 0.90 |
| Razykov, 2013 | 2013 | 345 | 9 | 249 | 0.69 | 0.75 |
| Vohringer, 2013 | 2013 | 190 | 55 | 101 | 0.93 | 0.77 |
| Zhang, 2013 | 2013 | 68 | 9 | 41 | 0.53 | 0.80 |
| Twist, 2013 | 2013 | 360 | 78 | 180 | 0.98 | 0.64 |
| Chagas, 2013 | 2013 | 84 | 19 | 54 | 1.00 | 0.83 |
| Akena, 2013 | 2013 | 91 | 10 | 71 | 0.91 | 0.89 |
| Santos, 2013 | 2013 | 196 | 19 | 145 | 0.76 | 0.85 |
| McGuire, 2013 | 2013 | 100 | 9 | 75 | 1.00 | 0.82 |
| Gelaye, 2014 | 2014 | 923 | 86 | 590 | 0.53 | 0.78 |
| Beraldi, 2014 | 2014 | 116 | 6 | 94 | 0.86 | 0.86 |
| Cholera, 2014 | 2014 | 397 | 38 | 292 | 0.81 | 0.83 |
| Fiest, 2014 | 2014 | 169 | 17 | 126 | 0.74 | 0.86 |
| Hyphantis, 2014 | 2014 | 349 | 78 | 236 | 0.82 | 0.93 |
| Kiely, 2014 | 2014 | 822 | 23 | 721 | 0.70 | 0.91 |
| Lambert, 2015 | 2015 | 147 | 15 | 103 | 0.71 | 0.82 |
| Amoozegar, 2017 | N/A* | 203 | 40 | 122 | 0.82 | 0.79 |
| Turner, unpublished | N/A* | 51 | 2 | 43 | 0.50 | 0.91 |

*: studies with no recollection date were only used for the BREM using all studies.
