# Supplementary material for: A case study of an individual participant data meta-analysis of diagnostic accuracy showed that prediction regions represented heterogeneity well
Source: Sci Rep. 2023 Jun 7;13:9275. doi: 10.1038/s41598-023-36129-w (PMC10247712; doi:10.1038/s41598-023-36129-w)
Supplement: Supplementary file 2 — Supplementary Information. [file 41598_2023_36129_MOESM2_ESM.docx]

**The Bivariate Random Effects Model (BREM)**

The BREM assumes that the outcome of the screening test has an unknown probability of being positive or negative depending on whether the participant truly has the disease. These probabilities correspond to the sensitivity and specificity of the test. For each study, the estimate for the logistic transform of these probabilities is assumed to be the sum of an overall mean logit-probability and a study-specific random effect. These random effects (one for sensitivity and one for specificity) are assumed to come from a joint normal distribution.

To determine the outcome of the PHQ-9 screening, only the previously mentioned cutoff score of greater than or equal to 10 was used. Let $y_{s,i}^{(0)}$ be the dichotomous outcome of the screening test for the $i$-th patient in the $s$-th study who does not have major depression ($y_{s,i}^{(0)}$ will equal one for a positive screen and zero for a negative screen). Analogously, let $y_{s,j}^{(1)}$ be the outcome of the test for the $j$-th patient in the $s$-th study who truly has major depression. The BREM is constructed as follows:

$$y_{s,i}^{\left( 0 \right)}\sim\text{Bernoulli}\left( p_{s,i}^{\left( 0 \right)} \right)$$

$$\text{logit}\left( p_{s,i}^{\left( 0 \right)} \right)=\mu_{s}^{\left( 0 \right)}=\mu^{(0)}+u_{s}^{(0)}$$

$$y_{s,j}^{\left( 1 \right)}\sim\text{Bernoulli}\left( p_{s,j}^{\left( 1 \right)} \right)$$

$$\text{logit}\left( p_{s,j}^{\left( 1 \right)} \right)={\mu_{s}^{\left( 1 \right)}=\mu}^{(1)}+u_{s}^{(1)}$$

$$\boldsymbol{u}_{s}=\binom{u_{s}^{(0)}}{u_{s}^{(1)}}\mathcal{\sim N}\left( \boldsymbol{0}, \boldsymbol{\Sigma} \right)\boldsymbol{;}\boldsymbol{\Sigma}\boldsymbol{=}\left( \begin{matrix} \tau_{0}^{2} & \tau_{0}\tau_{1}\rho_{\tau} \\ \tau_{0}\tau_{1}\rho_{\tau} & \tau_{1}^{2} \end{matrix} \right)$$

In this analysis, the measures of interest are the false positive rate (FPR), equal to 1-specificity, and the sensitivity or true positive rate (TPR). From the model described above the estimates for the pooled logit(FPR) and logit(TPR) are $\hat{\mu}^{(0)}$ and $\hat{\mu}^{(1)}$ respectively. Estimates for the between-study variance of the logit-transformed parameters are $\hat{\tau}_{0}^{2}$ and $\hat{\tau}_{1}^{2}$ respectively, and $\hat{\rho}_{\tau}$ is the estimated correlation.

*Constructing confidence and prediction ellipses*

In the univariate case, the conventional way to construct a $\left( 1-\alpha\right)100\%$ confidence interval for an estimated measure $\hat{\theta}$ (here the TPR or FPR) is:

$$\hat{\theta}\pm\mathcal{z}_{\frac{\alpha}{2}}\hat{\sigma}_{\theta}$$

where $\mathcal{z}_{\frac{\alpha}{2}}$ is the upper $\frac{\alpha}{2}$-th quantile of the standard normal distribution and $\hat{\sigma}_{\theta}$ is the estimated standard error of $\hat{\theta}$.

Analogously, a $\left( 1-\alpha\right)100\%$ prediction interval is given by:

$$\hat{\theta}\pm\mathcal{z}_{\frac{\alpha}{2}}\sqrt{\hat{\sigma}_{\theta}^{2}+\hat{\tau}^{2}}$$

where $\hat{\tau}^{2}$ is the estimate for the between-study variance component.

From a frequentist perspective, if a $\left( 1-\alpha\right)100\%$ confidence interval for a measure $\theta$ is constructed from sampling a population, then it has a $\left( 1-\alpha\right)$ probability of containing the true value of the measure. In contrast, a $\left( 1-\alpha\right)100\%$ prediction interval is concerned with covering the measure estimated from a new sample with probability $(1-\alpha)$ by characterizing its sampling distribution from the estimated population distribution.

In a bivariate case, we construct confidence or prediction regions in the two-dimensional parameter space. In the case of correlated measures, such as FPR and TPR, the shape of these regions will be elliptical.

Let $\hat{\boldsymbol{\mu}}$ be the column-vector of mean estimates for the two measures of interest calculated from $n$ observations:

$$\hat{\boldsymbol{\mu}}=\left[ \begin{aligned} \hat{\mu}^{(0)} \\ \hat{\mu}^{(1)} \end{aligned} \right]$$

Then let $\boldsymbol{\mu}$ be the vector of true means for these measures:

$$\boldsymbol{\mu}=\left[ \begin{aligned} \mu^{(0)} \\ \mu^{(1)} \end{aligned} \right]$$

And suppose that $\hat{\boldsymbol{\Psi}}$ is the estimated variance-covariance matrix for these fixed effects:

$$\hat{\boldsymbol{\Psi}}=\left[ \begin{matrix} \hat{\sigma}_{\mu^{(0)}}^{2} & \hat{\sigma}_{\mu^{(0)}}\hat{\sigma}_{\mu^{(1)}}\hat{\rho}_{\mu} \\ \hat{\sigma}_{\mu^{(1)}}\hat{\sigma}_{\mu^{(0)}}\hat{\rho}_{\mu} & \hat{\sigma}_{\mu^{(1)}}^{2} \end{matrix} \right]$$

The conventional approach, as described by Chew (see reference [7] in main manuscript), for constructing a $\left( 1-\alpha\right)100\%$ confidence region for $\boldsymbol{\mu}$ is via the quadratic form:

$$\left( \hat{\boldsymbol{\mu}}\boldsymbol{-\mu} \right)^{\top}{\hat{\boldsymbol{\Psi}}}^{-1}\left( \hat{\boldsymbol{\mu}}\boldsymbol{-\mu} \right)\boldsymbol{=}\chi_{\alpha}^{2}(2)$$

$$\left( \left[ \begin{aligned} \hat{\mu}^{\left( 0 \right)} \\ \hat{\mu}^{\left( 1 \right)} \end{aligned} \right]-\left[ \begin{aligned} \mu^{\left( 0 \right)} \\ \mu^{\left( 1 \right)} \end{aligned} \right] \right)^{\top}\left[ \begin{matrix} \hat{\sigma}_{\mu^{(0)}}^{2} & \hat{\sigma}_{\mu^{(0)}}\hat{\sigma}_{\mu^{(1)}}\hat{\rho}_{\mu} \\ \hat{\sigma}_{\mu^{(1)}}\hat{\sigma}_{\mu^{(0)}}\hat{\rho}_{\mu} & \hat{\sigma}_{\mu^{(1)}}^{2} \end{matrix} \right]^{-1}\left( \left[ \begin{aligned} \hat{\mu}^{\left( 0 \right)} \\ \hat{\mu}^{\left( 1 \right)} \end{aligned} \right]-\left[ \begin{aligned} \mu^{\left( 0 \right)} \\ \mu^{\left( 1 \right)} \end{aligned} \right] \right)=\chi_{\alpha}^{2}(2)$$

Where $\chi_{\alpha}^{2}(2)$ is the upper $\alpha$-th quantile of the chi-squared distribution with two degrees of freedom. In the equations above, it is important to highlight that every parameter has been estimated except for the components of $\boldsymbol{\mu}$. These are used as the coordinate axes in the parameter space the confidence and prediction regions are plotted in.

Similarly, to construct a prediction region for a new mean measure vector $\boldsymbol{\mu}^{\boldsymbol{*}}$:

$$\frac{1}{n+1}\left( \boldsymbol{\mu}^{*}-\hat{\boldsymbol{\mu}} \right)^{\top}{\hat{\boldsymbol{\Psi}}}^{-1}\left( \boldsymbol{\mu}^{*}-\hat{\boldsymbol{\mu}} \right)\boldsymbol{=}\chi_{\alpha}^{2}\left( 2 \right)$$

To account for uncertainty in the components of $\hat{\boldsymbol{\Psi}}$, as proposed by Chew, the equation for the ellipse will use a statistic from Hotelling’s $T^{2}$ distribution, the multivariate analogue of Student’s $t$ distribution, transformed into a Fisher-Snedecor $F$ distribution as follows:

$$T^{2}\left( 2, n-2 \right)=\frac{2\left( n-1 \right)}{n-2}F(2, n-2)$$

The proposed method for constructing a corrected $\left( 1-\alpha\right)\%$ prediction region for the new mean measure $\boldsymbol{\mu}^{*}$ is:

$$\frac{1}{n+1}\left( \boldsymbol{\mu}^{*}-\hat{\boldsymbol{\mu}} \right)^{\top}{(\hat{\boldsymbol{\Psi}}+\hat{\boldsymbol{\Sigma}})}^{-1}\left( \boldsymbol{\mu}^{*}-\hat{\boldsymbol{\mu}} \right)\boldsymbol{=}\frac{2\left( n-1 \right)}{n-2}F_{\alpha}(2, n-2)$$

Where $\hat{\boldsymbol{\Sigma}}$ is the estimated variance-covariance matrix for the random effects in the model and $F_{\alpha}(2, n-2)$ is the upper $\alpha$-quantile of the $F$ distribution with $2$ and $n-2$ degrees of freedom.
